# Hemimegalencephaly: A rare congenital malformation of cortical development

**DOI:** 10.1002/ccr3.5238

**Published:** 2021-12-18

**Authors:** Vikash Jaiswal, Muhammad Hanif, Zouina Sarfraz, Gaurav Nepal, Sidra Naz, Dattatreya Mukherjee, Samir Ruxmohan

**Affiliations:** ^1^ Larkin Community Hospital South Miami Florida USA; ^2^ Rani Primary Health Care Centre Biratnagar Nepal

**Keywords:** congenital, convulsions, hemimegalencephaly, seizures, unilateral megalencephaly

## Abstract

Hemimegalencephaly is a rare congenital malformation of cortical development usually associated with developmental delay and refractory epilepsy that sooner or later require hemispherectomy.

## Graphical abstract

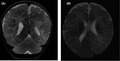

## 1 INTRODUCTION

Hemimegalencephaly (HME) is a rare cortical malformation marked by the enlargement of one cerebral hemisphere. The pathogenesis is not fully understood although there have been suggestions of genetic links and an association with mTOR (mammalian target of rapamycin) pathway mutations.^1^ The most common presentation of HME is seizures in the patient, although other neurological manifestations like developmental delays, hemianopia, and motor weakness have been reported in current literature. The seizures associated with HME are rarely controlled with the use of anti‐epileptic drugs (AEDs). Hemispherectomy is an effective method of treatment for multi‐drug‐resistant seizures. We present a rare case of a 22‐month‐old male child with HME presenting with seizures, which was controlled with AEDs and was later free of breakthrough seizures attacks.

## 2 CASE PRESENTATION

A 22‐month‐old male child with a past history of HME, infantile spasms, developmental delay, and seizures presented to the emergency department with an increased seizures frequency and character for 2 days. Previously, the patient's seizure episode lasted for a few seconds and the semiology was consistent with staring out episodes and limping. However, in the last 2 days, the semiology of the epileptic episodes had changed, occurring for about 1 min and consisted of falling to the floor associated with abnormal jerking movements, incontinence and up rolling of eyes. There was no history of trauma, fever, rashes, vomiting, diarrhea, upper respiratory tract infections, and exposure to any drugs. There was no family history of seizures and neurocutaneous disorders.

On physical examination, his weight was 12.3 kg, blood pressure was 110/63 mm of Hg, pulse rate was 116/min, temperature was 98.9 F, SpO2 was 100% at room air and respiratory rate was 23/min. There was no skin features suggestive of neurocutaneous disorders. Systemic examination findings were unremarkable. Baseline investigations along with metabolic profile results were within normal limit (Table 1).

**TABLE 1:** Baseline investigations of the patient

Test	Result
Hemoglobin	12.4 g/dl
Total leukocyte count	8.8(×10^9^/L)
Red blood cells count	4.3 (×10^12^/L)
Platelets	290 × 10^9^/L
Prothrombin time	12 s (12 s control)
Activated partial thromboplastin time	28 s (28 s control)
Alanine aminotransaminase (ALT)	32 U/L
Aspartate aminotransaminase (AST)	28 U/L
Alkaline phosphatase	43 U/L
Total bilirubin	0.2 mg/dl
Blood urea	21 mg/dl
Creatinine	0.4 mg/dl
Blood glucose	6 mmol/L
Sodium	137.2 mEq/L
Potassium	3.92 mEq/L
Chloride	103 mEq/L
Serum calcium	9.8 mg/dl
Total cholesterol	115 mg/dl
Triglycerides	109 mg/dl
High‐density lipids	41 mg/dl
Low‐density lipids	170 mg/dl

**FIGURE 1:**
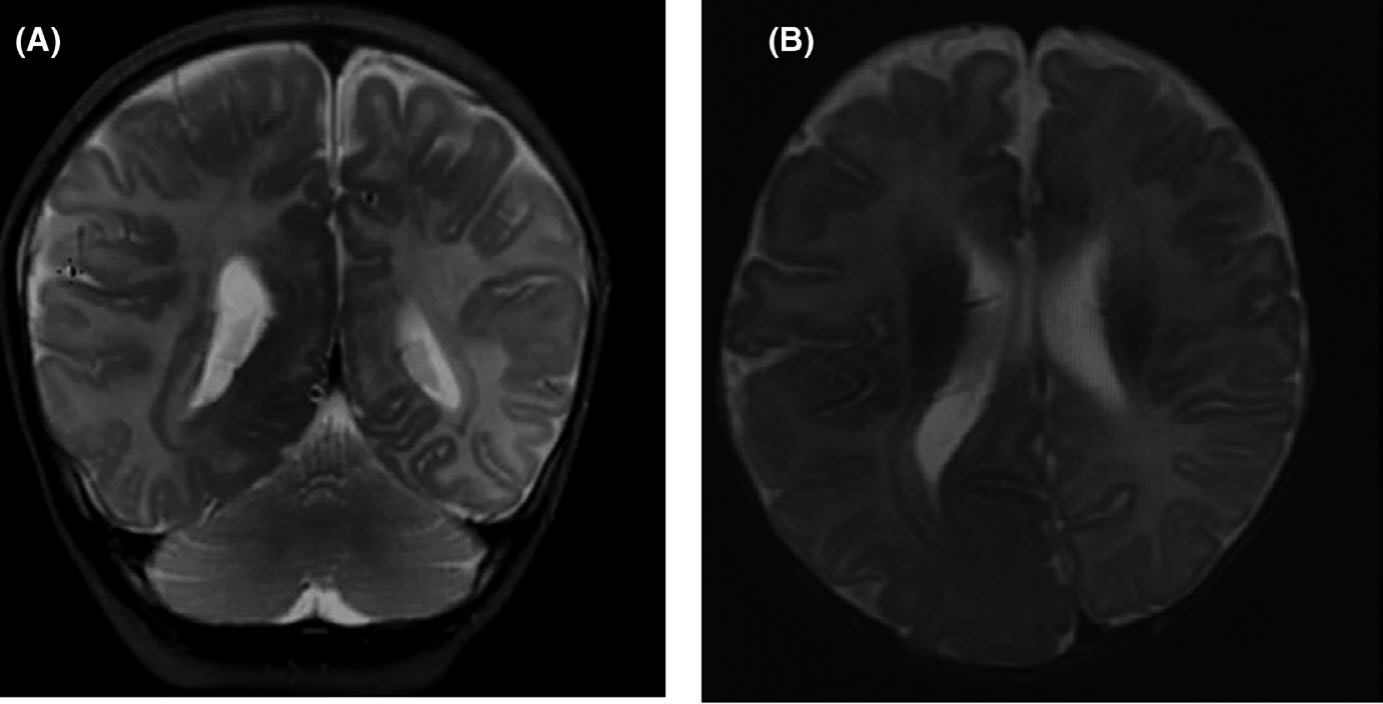
T2 weighted magnetic resonance images (A: coronal, B: axial) demonstrating typical features of hemimegalencephaly, including enlarged right hemisphere with straightening of frontal horn, ventricular dysmorphism, white matter changes with herniation toward the left cerebral hemisphere, and enlargement of the right lateral ventricle. The gyri are larger with effacement of the sulci are seen in the right hemisphere in comparison to the left. The head circumference is larger for the age group

The patient was given a bolus dose of levetiracetam (20 mg/kg) at the emergency department and no seizure activity was noted under the observation of the nursing team. He commenced on topiramate 30 mg twice a day, clonazepam 0.25 mg twice a day, injectable lorazepam as needed in case of seizure activity, and acetaminophen as needed. An electroencephalogram (EEG) was performed, which showed poor regional organization and lack of a posterior dominant rhythm as well as continuous epileptiform activity in the right occipital‐temporal‐parietal region. A magnetic resonance imaging (MRI) of the brain showed asymmetric enlargement of the right cerebral hemisphere with hypermyelination, mild ventriculomegaly, and displaced posterior falx. These findings were mostly consistent with hemimegalencephaly (Figure 1). The patient was admitted to the pediatric ward and was kept under close observation for the next 48 h. No seizure activity was noted under observation and he was discharged home on AEDs. At 6 months follow‐up, the patient was doing well and free of breakthrough seizures attacks.

## 3 DISCUSSION

Hemimegalencephaly is a rare genetic condition and cortical malformation associated with seizures and is marked by the enlargement of one cerebral hemisphere.^2^ HME is broadly divided into three types: isolated, syndromic, and total. Isolated HME has no association with hemicorporal hypertrophy, while syndromic HME has an association with ipsilateral hemicorporal hypertrophy. On the other hand, total HME, which is also the least common form, is associated with enlargement of the cerebellum and brainstem.^3^

The pathogenesis of the disease is thought to be characterized by abnormal activation of the mTOR signaling pathway^2^ because mTOR inhibitors such as everolimus play a key role in the treatment of hemimegalencephaly. Occasionally, the seizures associated with HME are difficult to control with AEDs and hemispherectomy is an effective treatment option for drug‐resistant seizures.^4^ Hereditary pathogenesis hypotheses and modern histopathology offer a basic explanation of the complex malformation. Imperative findings include the key disruption in neuronal lineage, division, and proliferation, disruption of gene expression, and the untimely initiation of radial neuroblast migration.^3^

Neurocutaneous conditions such as neurofibromatosis, epidermal nevus syndrome, Ito's hypomelanosis, and Klippel‐Trenaunay‐Weber syndrome may induce hemimegalencephaly, wherein serious and drug‐resistant epilepsy dominates the clinical image. Macrocrania, moderate/severe development delays, unilateral motor deficiency, and hemianopia are other typical findings, which have been reported in the literature.^5^ The EEG typically demonstrates several irregular patterns, the most prominent of which are inhibition bursts and/or hemi‐hypsarrhythmia. While standard observations on neuroimaging and histologic investigations (enlarged hemisphere, malformed ventricular system, modification of regular gyration) are common, creating a differential diagnosis with other disorders of neuronal and glial proliferation may be particularly challenging.^5^

In our case, a 22‐month‐old boy was admitted because of acute seizure onset and the MRI brain image showed a right‐sided enlargement of the cerebral hemisphere with hypermyelination and ventriculomegaly. The patient's seizures were controlled with a modified AED regimen. However, in many cases, AEDs are unsuccessful, where hemispherectomy is the treatment of choice. This surgical procedure, although an effective method for drug‐resistant seizure, has adverse effects such as, postoperative bleeding, surgical infections, meningitis, which can be prevented by prescribing steroids and hydrocephalus (which may be early or delayed). In similar cases, multi‐disciplinary care is recommended particularly in the pediatric age group with a focus on the neurological wellbeing of the patient.

## 4 CONCLUSION

Hemimegalencephaly, a rare condition, usually presents with a seizure and has been found resistant to numerous AEDs. Hemispherectomy, which has many early and delayed complications, is the treatment of choice in AED‐resistant seizures. The seizures in our patient was controlled with AEDs and was later free of breakthrough seizures attacks. A multi‐disciplinary care plan is required to improve the neurological prognosis.

## CONFLICT OF INTEREST

The case report was presented at the 146^th^ Annual Meeting of American Neurological Association.

## AUTHOR CONTRIBUTIONS

VJ, MH, DM, and SN contributed to the collection of case information, writing of the manuscript, and manuscript revision. GN, ZS, and SR were involved in revising the manuscript critically for important intellectual content. All authors approved the final version.

## ETHICAL APPROVAL

This study did not include experiments on animals or humans.

## CONSENT

Written informed consent was obtained from the patient's guardians for publication of this case report and any accompanying images. A copy of the written consent is available for review by the Editor‐in‐Chief of this journal.

## Data Availability

Not applicable.
